# TNF-α enhances the effect of TGF-β on Gli2 expression in the KG-1 leukemic cell line

**DOI:** 10.3892/etm.2014.1743

**Published:** 2014-05-28

**Authors:** ZHE LI, BIN LI, JING PAN, JIEPING JIN

**Affiliations:** Department of Hematology, First Affiliated Hospital of Liaoning Medical College, Jinzhou, Liaoning 121001, P.R.China

**Keywords:** Gli family zinc finger 2, transforming growth factor-β, tumor necrosis factor α, specific inhibitor of smad3

## Abstract

The Hedgehog (Hh) signaling pathway regulates a variety of tumor-related diseases, including leukemia. The present study aimed to determine whether there was an interaction between the Hh signaling pathway and transforming growth factor (TGF)-β in the KG-1 cell line. KG-1 cells were treated with TGF-β, tumor necrosis factor (TNF)-α and specific inhibitor of smad3 (SIS3). The expression level of Gli family zinc finger 2 (Gli2) was detected by quantitative polymerase chain reaction (qPCR) and western blot analyses. The results revealed that TGF-β significantly decreased the expression level of Gli2 in KG-1 cells, and that TNF-α and TGF-β together further reduced Gli2 expression in KG-1 cells. SIS3 inhibited the effect of TGF-β. These results suggest that Gli2 expression in KG-1 cells is suppressed by TGF-β in a Smad3-dependent manner, TNF-α can enhance the effect of TGF-β on Gli2 expression and that this occurs independently of Hh receptor signaling.

## Introduction

The Hedgehog (Hh) signaling pathway is linked to cell growth and differentiation. It is involved in embryonic pattern formation and adult tissue homeostasis (1). Recently, the Hh signaling pathway has been revealed to be dysregulated in several human malignancies and to be critical for the maintenance and expansion of malignant stem cells (2). Gli family zinc finger (Gli) transcription factors constitute the final effectors of the Hh signaling pathway. In a number of tumors, including those of the pancreas, prostate, skin or lungs, the ectopic activation of Gli proteins has been linked to tumorigenesis (3). There has also been evidence suggesting additional, noncanonical mechanisms of Gli activation (3). Certain studies have suggested that in normal fibroblasts and keratinocytes, as well as in various cancer cell lines, the Gli transcription factors are not solely regulated by Hh/smoothened (Smo) signaling, but also by other pathways, including transforming growth factor (TGF)-β signaling. TGF-β has been shown to induce Gli2 expression in a Smad3-dependent manner in various cell types and this effect was demonstrated to be independent from the patched (Ptch)/Smo axis (4).

In hematopoiesis, Hh family members play an important role in the regulation of stem/progenitor cell expansion *in vitro* and *in vivo* (5). Components of Hh signaling have been detected in several leukemic cell lines including: Ptch and Smo, which are expressed in Jurkat cells (6); sonic hedgehog (Shh) and Gli1, in HL-60 and KG-1 cells (7); and Gli2 in U937 and HL60 cells (8). Similarly to the Hh members, TGF-β plays an important role in regulating the balance between proliferation and differentiation in hematopoietic cells (9,10).

With the aim of identifying a new target for leukemia treatment, in the present study it was hypothesized that there is also cross-communication between the Hh signaling pathway and TGF-β in leukemic cells. In the current study, the capacity of TGF-β for modulating the expression of the Hh signaling molecule Gli2 in the KG-1 human myeloid leukemia cell line was examined. Targeting the interaction between Hh and TGF-β signaling may provide novel therapeutic opportunities for leukemia treatment.

## Materials and methods

### Cell cultures and reagents

KG-1 human myeloid leukemia cells were donated for use in the present study by the Institute of Hematology of the Chinese Academy of Medical Sciences (Tianjin, China). They were cultured in Iscove’s modified Dulbecco’s medium (IMDM, Solarbio, Beijing, China) supplemented with 20% heat-inactivated fetal calf serum (FCS), 0.1% penicillin (100 U/ml) and streptomycin (100 mg/ml), at 37°C in a humidified atmosphere of 5% CO_2_. When the cells were in the logarithmic growth phase, the KG-1 cells were seeded on starving medium (containing 2.5% FCS) at the same cell density of 2×10^5^ cells/ml. They were treated with human recombinant TGF-β1; 5 ng/ml; referred to as TGF-β), tumor necrosis factor (TNF)-α; 5 ng/ml) and/or specific inhibitor of smad3 (SIS3; 5 μM) for various time periods. TGF-β1 and TNF-α were purchased from Peprotech Inc. (Rocky Hill, NJ, USA). SIS3 was purchased from Merck KGaA (Darmstadt, Germany). The monoclonal antibody anti-Gli2 was purchased from Santa Cruz Biotechnology Inc. (Santa Cruz, CA, USA). The monoclonal antibody anti- TGF-βRI and anti-TGF-βRII were purchased from Bioss Biotechnology Inc. (Bioss, Beijing, China).

### Reverse transcription (RT) and quantitative (q) polymerase chain reaction (PCR)

The total RNA was extracted using an RNeasy mini kit (Qiagen, Hilden, Germany). RNA (1 μg) was reverse-transcribed using SuperScript™II (Invitrogen Life Technologies, Takara, Japan). Reverse transcription was carried out on genomic DNA-free RNA using random primers. The RT-PCR procedures were carried out on a GeneAmp PCR System 9700 (Thermo Fisher Scientific, Waltham, MA, USA). The qPCR was subsequently performed using SYBR Green PCR Core reagents and a Rotor-Gene 6000 real-time rotary analyzer (Corbett Life Science, Sydney, Australia) to quantify the steady-state levels of Gli2 mRNA. The expression level of the housekeeping gene ABL was used as a control. The process was repeated three times for each sample. The final results were compared by the comparative ΔΔ Ct method. The primer pairs for Gli2 (5′-TGGCCGCTTCAGATGACAGATGTTG-3′ and 5′-CGTTAGCCGAATGTCAGCCGTGAAG-3′) and ABL (5′-CGAGAGCCTGGCCTACAACAA-3′ and 5′-CTAGCA GCTCATACACCTGGGACA-3′) were designed and synthesized by Takara Bio, Inc. (Shiga, Japan). PCR amplification was carried out using 45 cycles of 95°C for 60 sec, 95°C for 10 sec and 60°C for 30 sec.

### Western blot analysis

At the end of the incubation period, cells were washed twice in ice-cold phosphate-buffered saline (PBS), following centrifugation at 200 g and 4°C. The cells were immediately transferred to ice-cold lysis buffer (SunShineBio, Nanjing, China) and phenylmethylsulfonyl fluoride (PMSF) was added. The solution was shaken at 4°C for 30 min. Following this, the cellular extracts were collected by centrifugation at 16,000 g for 5 min at 4°C and the samples were immediately frozen at −80°C. The samples were homogenized with a 4-(2-hydroxyethyl)-1-piperazineethanesulfonic acid (HEPES) buffer solution (Gibco, Carslbad, CA, USA; 100 μg/lane), separated by 10% sodium dodecyl sulfate polyacrylamide gel electrophoresis (SDS-PAGE) and blotted onto polyvinylidene fluoride (PVDF) membranes. Subsequently, the membranes were blocked with Tris-buffered saline and Tween 20 (TBST) containing 5% skimmed milk. Gli2, TGF-βRI or TGF-βRII protein content was detected with an anti-Gli2, anti-TGF-βRI or anti-TGF-βRII antibody. An antibody directed against β-actin (Santa Cruz) was used to verify the equal protein content in each sample. Following washing with TBST, the membranes were incubated with goat anti-rabbit IgG (H+L)/TRITC secondary antibodies (Santa Cruz). The membranes were detected with an electrochemiluminescence (ECL) Western blotting system (Alpha Diagnostic International, San Antonio, TX, USA).

### Statistical analysis

The gene expression levels in the KG-1 cell line were compared by one way analysis of variance (ANOVA). P<0.05 was considered to indicate a statistically significant difference. Results obtained from multiple experiments are reported as the mean ± standard error of the mean.

## Results

### TGF-β affects the expression level of Gli2 in the KG-1 cell line, while TNF-α does not

**Figure 1:**
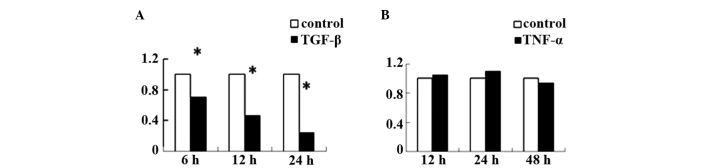
Effect of transforming growth factor β (TGF-β) or tumor necrosis factor α (TNF-α) alone on the expression level of Gli family zinc finger 2 (Gli2) mRNA in the KG-1 cell line. (A) KG-1 cells were divided into two treatment groups: control group (no treatment) and the TGF-β group which received 5 ng/ml transforming growth factor β1 (TGF-β1) for 6, 12 and 24 h. (B) KG-1 cells were divided into two treatment groups: control group (no treatment) and the TNF-α group which received 5 ng/ml TNF-α for 12, 24 and 48 h. The total mRNA in the KG-1 cells was extracted and quantitative PCR was performed. ^*^P<0.05, TGF-β1 group vs. control group.

To determine whether TGF-β or TNF-α affected the expression level of Gli2, KG-1 cells were incubated with TGF-β, with TNF-α or without either (control). A significant reduction in the expression level of Gli2 mRNA in response to TGF-β was observed in the KG-1 cells (Fig. 1A). This reduction was observed following 6 h of treatment and lasted for a minimum of 24 h, with the TGF-β values significantly lower than those in the control group during the experimental period. As shown in Fig. 1B, TNF-α did not demonstrate a significant effect on the expression level of Gli2 mRNA in the KG-1 cells.

### TNF-α increases TGF-β type I receptor (TGF-βRI) and TGF-β type II receptor (TGF-βRII) protein expression levels in KG-1 cells

**Figure 2:**
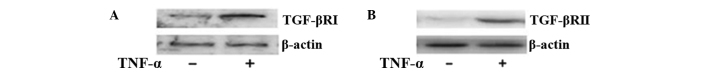
Tumor necrosis factor (TNF)-α increases transforming growth factor β type I receptor (TGF-βRI) and transforming growth factor β type II receptor (TGF-βRII) protein expression levels in KG-1 cells. KG-1 cells were treated with 5 ng/ml TNF-α (+ group) or not treated as the control (− group) for 24 h. The total proteins in the KG-1 cells were extracted and a western blot analysis was performed.

KG-1 cells were incubated with TNF-α in the TNF-α (+) group, or were untreated in the control (−) group for 24 h. Fig. 2 show that the TGF-βRI and TGF-βRII protein expression levels in the KG-1 cells were significantly higher in the TNF-α (+) group than in the control (−) group.

### TGF-β strongly reduces the expression level of Gli2 when KG-1 cells are incubated with TGF-β and TNF-α in combination

**Figure 3:**
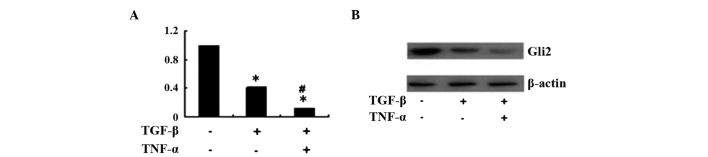
Effect of treatment with transforming growth factor β (TGF-β) with or without tumor necrosis factor (TNF)-α on the expression level of Gli family zinc finger 2 (Gli2) in KG-1 cells. KG-1 cells were treated with: no treatment as a control (−), 5 ng/ml transforming growth factor β1 (TGF-β1; +) or 5 ng/ml TGF-β1+5 ng/ml TNF-α (+), respectively for 24 h. (A) The total mRNA in the KG-1 cells was extracted and quantitative PCR was carried out. (B) The total proteins in the KG-1 cells were extracted and western blot analyses were performed. ^*^P<0.05, treatment group (+) vs. control (−) group; ^#^P<0.05, TGF-β1 + TNF-α group vs. TGF-β1 group.

To investigate whether a combination of TGF-β and TNF-α is able to influence the expression level of Gli2 in KG-1 cells, the KG-1 cells were cultured with TGF-β, in the presence or absence of TNF-α for 24 h. As shown in Fig. 3, the expression of Gli2 at the mRNA and protein levels in the TGF-β + TNF-α group was much lower than the expression of Gli2 in the TGF-β group and the control (−) group. The expression level of Gli2 in the TGF-β group was lower than that in the control (−) group.

### Suppression of Gli2 expression by TGF-β is Smad3-dependent

**Figure 4:**
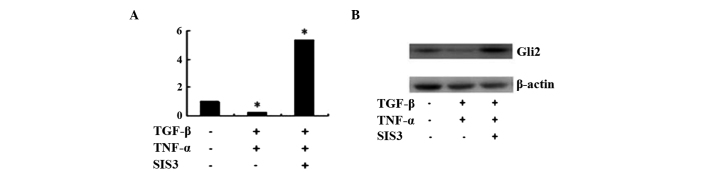
Effect of treatment with transforming growth factor (TGF)-β and tumor necrosis factor α (TNF-α) with or without smad3 inhibitor (SIS3) on the expression level of Gli family zinc finger 2 (Gli2) in KG-1 cells. KG-1 cells were treated with: no treatment as a control (−), 5 ng/ml transforming growth factor β1 (TGF-β1) + 5 ng/ml TNF-α or 5 ng/ml TGF-β1 + 5 ng/ml TNF-α + 5 uM SIS3, respectively for 24 h. (A) The total mRNA in the KG-1 cells was extracted and quantitative PCR was performed. (B) The total proteins in the KG-1 cells were extracted and western blot analyses were carried out. ^*^P<0.05, treatment group (+) vs. control (−) group.

To determine whether the suppression of Gli2 expression by TGF-β was Smad3-dependent, SIS3 (11), a specific inhibitor of Smad3, was used in combination with TGF-β and TNF-α to treat the KG-1 cells. Fig. 4 shows that the expression of Gli2 in the KG-1 cells at the mRNA and protein levels decreased compared with the levels in untreated cells when they were treated with TGF-β + TNF-α, and increased when they were treated with TGF-β + TNF-α + SIS3.

### SIS3 increases the expression level of Gli2 in KG-1 cells

**Figure 5:**
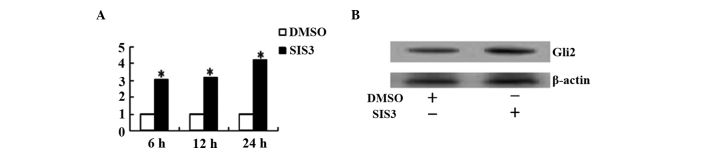
Specific inhibitor of Smad 3 (SIS3) is able to increase the expression of Gli family zinc finger 2 (Gli2) in KG-1 cells at the protein and mRNA levels. The KG-1 cells were treated with 5 μM SIS3 or the same amount of control solution (dimethyl sulfoxide, DMSO) for 6, 12 and 24 h. (A) The total mRNA in the KG-1 cells was extracted and quantitative PCR was performed. (B) The total proteins in the KG-1 cells were extracted and western blot analyses were carried out. ^*^P<0.05, SIS3 group vs. DMSO group.

To determine whether the TGF-β secreted by the KG-1 cells themselves is able to affect the expression level of Gli2, the KG-1 cells were pretreated with SIS3 or a control (dimethyl sulfoxide, DMSO). Fig. 5 shows that the expression of Gli2 at the mRNA and protein levels in the KG-1 cells treated with SIS3 was significantly higher than the expression in those treated with DMSO.

## Discussion

Hh signaling is critical in vertebrate development, patterning, and cell fate induction (12). Deregulation of Hh signaling is associated with different forms of human cancer (2). The Hh signaling pathway comprises Hh ligands, Ptch and Smo receptors, and Gli zinc-finger protein transcription factors (13). The secreted protein Hh acts by binding to its receptor Ptch, which then ceases to inhibit another transmembrane protein, Smo. This activates downstream cytoplasmic transcription factors. In *Drosophila* these are the Ci proteins and the mammalian homologues of these are the Gli proteins (1). This downstream signaling initiates the entry of cells into the cell cycle (14) in order to maintain the self-renewal of stem cells in various tissues (15), inhibit apoptosis (16), modulate tissue polarity (17) and regulate the differentiation of tissue stem cells (18). Evidence has suggested that the Hh signaling pathway is prominent in cancer stem cells (19). Recently, potential methods of inhibiting the Hh signaling pathway have been studied in order to develop a new class of possible therapeutics for cancer treatment (20,21).

There are three related Gli proteins: Gli1, Gli2 and Gli3 (22). Gli2 functions upstream of Gli1 and is the primary mediator of Hh signaling (23), inducing Gli1 expression via direct binding to its promoter region (24). Gli3 genes inhibit the activating functions of all coexpressed Gli genes (25).

Similarly to Hh members, as a family of growth factors involved in various essential physiological processes, TGF-β plays a complex role as a mediator in inflammation, tissue repair, angiogenesis, and the regulation and differentiation of cell growth (26). TGF-β binds to a heteromeric cell-surface complex of type I (TGF-TβRI) and type II (TGF-TβRII) serine/threonine kinase receptors (27). Following ligand binding, TβRII recruits and activates TβRI, which phosphorylates Smad proteins. The receptor-associated Smad proteins, Smad2 and Smad3, heteromerize with Smad4 and later translocate into the nucleus and act as transcription factors to regulate target gene expression (28).

There is abundant data regarding the effect of TGF-β on leukemic cells lines, ranging from inhibition of proliferation and induction of differentiation, to altered expression of adhesion molecules and cytokine receptors, and the induction of apoptosis (29). Acting as an inhibitory autocrine factor in the proliferation of leukemia cells (30), TGF-β1 is a primary negative regulator of early hematopoiesis (31).

Hh receptor molecules are lost in several leukemia cell lines, including KG-1 cells; however, the transcription factor Gli2 is expressed (8). For these cells, the blockade of the autocrine loop in Hh signaling would not be available. Thus, Gli2 may be an alternative target for the treatment of acute myeloid leukemia (AML). One possible strategy is to use small interfering RNAs (siRNAs) that are specific to Gli2. However, efficient transfer of siRNAs into the hematopoietic cells is problematic (32). The present study revealed that TGF-β, alone or combined with TNF-α, was able to decrease the expression level of Gli2 in the KG-1 cell line, thus suggesting that TGF-β may be one of the signaling molecules that plays a key role in the regulation of the Gli2 gene in leukemia cell lines.

Treatment with TGF-β significantly affected the expression level of Gli2 in the KG-1 cells in the present study, whereas no significant differences in the expression levels of Gli2 were observed between the control and TNF-α groups of KG-1 cells (Fig. 1). A previous study has revealed that TNF-α is able to affect Shh through the nuclear factor κ-light-chain-enhancer of activated B cells (NF-κB) pathway (33), and that Shh is able to affect the expression level of Gli2. However, the Hh receptor molecules (Ptch and Smo) are lost in KG-1 cells (8). This may explain why TNF-α did not affect the expression level of Gli2 in the KG-1 cells. In the present study, when the KG-1 cells were treated with TNF-α, it was found that the TGF-βRI and TGF-βRII protein expression levels were increased in the KG-1 cells (Fig. 2). This result is consistent with the results of a previous study (34). Furthermore, the Gli2 levels in the KG-1 cells treated with TGF-β + TNF-α were different from those in the cells only treated with TGF-β (Fig. 3). The expression level of Gli2 in the KG-1 cells treated with TGF-β and TNF-α was lower than the level in those treated with TGF-β alone (Fig. 3). It was possible that the expression of TGF-β receptors in the KG-1 cells was increased by TNF-α, when enabled TGF-β to strongly decrease the expression of Gli2 expression.

The mechanism by which TGF-β decreases the expression level of Gli2 in KG-1 cells involves Smad2 and Smad3. By acting as a downstream signaling molecule for TGF-β, Smad3 contributes to the majority of Smad-dependent responses to TGF-β in adults. Smad2, however, is critical during embryogenesis (35,36). Specific inhibitor of Smad3 (SIS3) was used to inhibit the effects of TGF-β. Fig. 4 shows that SIS3 inhibited the effects of TGF-β + TNF-α, which otherwise decreased the expression levels of Gli2 in the KG-1 cells. Since Ptch and Smo are not expressed in KG-1 cells (8), this effect was independent from the Ptch/Smo axis. Thus, the suppression of Gli2 expression by TGF-β occurs in a Smad3-dependent manner.

The present study also investigated the effect of SIS3 on the expression level of Gli2 in KG-1 cells. As shown in Fig. 5, SIS3 significantly increased the expression level of Gli2 in the KG-1 cells. SIS3 is a specific inhibitor of Smad3, which has been shown in the present study to act via blocking the TGF-β signaling pathway. As this effect on Gli2 was observed without adding TGF-β to the KG-1 cells, this suggests that TGF-β secreted by the KG-1 cells is able to decrease Gli2 expression.

TGF-β has been revealed to have growth-enhancing and growth-inhibitory properties, the predominating effect being dependent on the particular cell type and the other growth factors present (37). The effect of TGF-β on KG-1 cells in the current study differed from a previous study which have reported that TGF-β increases the expression level of Gli2 in various human cell types, including normal fibroblasts and keratinocytes, as well as in various cancer cell lines (4). This variation may be due to the characteristics of the different cell lines and the other growth factors present.

In conclusion, the present study revealed that a combination of TGF-β and TNF-α decreased the expression level of Gli2 in KG-1 cell lines and that SIS3 inhibited the effect of TGF-β. The suppression of Gli2 expression was Smad3-dependent. These results demonstrate that TGF-β plays a role as a cytokine that may be capable of decreasing Hh signaling in certain types of leukemic cell lines. This may initiate the development of new approaches for the efficient therapeutic treatment of certain types of leukemia; however, further *in vivo* and *in vitro* studies are required.
